# Continued Selenium Biofortification of Carrots and Broccoli Grown in Soils Once Amended with Se-enriched *S. pinnata*

**DOI:** 10.3389/fpls.2016.01251

**Published:** 2016-08-23

**Authors:** Gary S. Bañuelos, Irvin S. Arroyo, Sadikshya R. Dangi, Maria C. Zambrano

**Affiliations:** Water Management Research Unit, San Joaquin Valley Agricultural Sciences Center, United States Department of Agriculture, Agricultural Research Service, Parlier, CAUSA

**Keywords:** selenium, biofortification, *Stanleya pinnata*, carrots, broccoli

## Abstract

Selenium (Se) biofortification has been practiced in Se-deficient regions throughout the world primarily by adding inorganic sources of Se to the soil. Considering the use of adding organic sources of Se could be useful as an alternative Se amendment for the production of Se-biofortified food crops. In this multi-year micro-plot study, we investigate growing carrots and broccoli in soils that had been previously amended with Se-enriched *Stanleya pinnata* Pursh (Britton) three and 4 years prior to planting one and two, respectively. Results showed that total and extractable Se concentrations in soils (0–30 cm) were 1.65 mg kg^-1^ and 88 μg L^-1^, and 0.92 mg kg^-1^ and 48.6 μg L^-1^ at the beginning of the growing season for planting one and two, respectively. After each respective growing season, total Se concentrations in the broccoli florets and carrots ranged from 6.99 to 7.83 mg kg^-1^ and 3.15 to 6.25 mg kg^-1^ in planting one and two, respectively. In broccoli and carrot plant tissues, SeMet (selenomethionine) was the predominant selenoamino acid identified in Se aqueous extracts. In postharvest soils from planting one, phospholipid fatty acid (PLFA) analyses showed that amending the soil with *S. pinnata* exerted no effect on the microbial biomass, AMF (arbuscular mycorrhizal fungi), actinomycetes and Gram-positive and bacterial PLFA at both 0–5 and 0–30 cm, respectively, 3 years later. Successfully producing Se-enriched broccoli and carrots 3 and 4 years later after amending soil with Se-enriched *S. pinnata* clearly demonstrates its potential source as an organic Se enriched fertilizer for Se-deficient regions.

## Introduction

Selenium biofortification of food crops has been practiced in Se-deficient regions of different countries by adding inorganic-Se containing fertilizers to soils, e.g., Finland (Alfthan et al., 2010), United Kingdom (UK) (Lyons, 2010), New Zealand (Curtin et al., 2006), and in China (Wu et al., 2015). In Finland, where the geochemical soil conditions are relatively uniform, two decades of supplementation of soils nationwide with fertilizers containing inorganic-Se has proven to be a safe and effective way of significantly increasing Se concentrations in most crops grown for human consumption (Alfthan et al., 2010). Similarly, Great Britain has undertaken efforts to develop soil amendment practices with inorganic-Se designed to increase dietary Se intake in the general population via the Se biofortification of food (Rayman, 2012). For this purpose, the successful use of inorganic-Se fertilizers is, however, strongly dependent on uniform physical soil conditions with consideration of soil types, soil redox potentials, soil pH (Hartfiel and Bahners, 1988), and the absence of high soil sulfate concentrations and elevated organic matter (Terry et al., 2000). Although Se-containing fertilizers can be effective for producing Se-enriched food and feed products, excessive bioaccumulation and downward migration of soluble inorganic Se under variable field conditions can occur, especially in lighter-textured soils. Hence, Se fertilization strategies must be carefully designed for non-uniform soil growing conditions or in soils with shallow groundwater.

Realistically, applying inorganic-Se accurately at low rates (e.g., 20 g ha^-1^) on an annual basis can be especially difficult for untrained growers under variable field soil conditions or in countries with less developed farming methods and with a diverse set of cropping systems. Moreover, losses of Se can occur through leaching of soluble inorganic-Se by excessive precipitation/irrigation or by the volatilization of Se (e.g., methyl-selenide) induced by microbial metabolic activities (Žnidarčič, 2011). Other researchers have incorporated agronomic or biotechnological methods to biofortify crops with Se (Lyons et al., 2004; White and Broadley, 2005, 2009; Hirschi, 2009; Bouis et al., 2011), including applying foliar applications of Se (Kápolna et al., 2012; Pezzarossa et al., 2012; Wang et al., 2013), or growing crops hydroponically in a Se-rich nutrient medium (Smoleń et al., 2014). The consideration of adding other sources of Se, e.g., organic-Se enriched fertilizers, could be useful as an alternative soil amendment to produce Se biofortified food crops. For this purpose, early amendment studies have been conducted with the addition of Se enriched plant materials to soils (Bañuelos et al., 1992; Ajwa et al., 1998), and most recently, in carrots and broccoli by adding Se-enriched hyperaccumulator plant material (e.g., *Stanleya pinnata*) to soils (Bañuelos et al., 2015). In all these studies, Se applied via organic sources was absorbed by the respective plant species. In this regard, earlier work by Martens and Suarez (1997) reported upon the changes of Se after the addition and decomposition of organic sources of Se – selenomethionine (SeMet) and selenocystine (SeCys_2_) – to the soil. They suggested that Se added as SeMet will not accumulate in soil due to its volatile loss from the soil as dimethyl selenide. Oftentimes, yeast is commonly used as a source of organic Se, in which Se is predominately found as SeMet. In contrast, the addition of seleniferous plant residues rich in SeCys_2_ will result in organic mineralization to inorganic Se forms in the soil and will eventually be available for plant uptake (Martens and Suarez, 1997). Compared to organic sources of SeMet, selenocystathionine (SeCyst) was the predominate form of Se applied via *S. pinnata* in a Se biofortification study conducted by Bañuelos et al. (2015) for one growing season. To the authors knowledge there are no studies that have reported on the continued availability of Se when added to soil in non-selenomethionine forms, e.g., SeCyst. Another question arises on whether Se-enriched plant material added to soil has an effect on the presence and participation of soil microorganisms. The presence, participation, or effect on soil microbes/microbial community in soils previously amended with Se-enriched material, e.g., *S. pinnata*, has not been investigated, although there are generally contradictory reports on interactive effects of soil Se on micro-organisms. For example, Chander and Joergensen (2007) reported no negative effects of Se on soil microbial biomass C and adenosine triphosphate (ATP) concentrations, which contradicts research findings by Frankenberger and Karlson (1994) who showed that microbial-induced volatilization of Se was a protection strategy to avoid toxicity in seleniferous environments. If the soil microorganisms play a role in nutrient cycling and organic matter decomposition (Dangi et al., 2014), they may also participate in transforming or incorporating organic forms of Se into plant available Se (Martens and Suarez, 1997). Similarly, Lindblom et al. (2013) reported that Se tolerant rhizosphere bacteria can boost Se accumulating ability in *Brassica juncea* (L.) Czern, as well as affect localization and speciation of Se in the roots of Se hyperaccumulators. More research is clearly needed on identifying presence of the microbial community in soils amended with Se, especially organic sources of Se, between different plant species (Ravit et al., 2006).

In this 2-year biofortification field study, we attempt to investigate the following with carrots and broccoli grown in soils previously amended (3–4 years ago) with organic Se from the Se-hyperaccumulator *S. pinnata*: (1) determine uptake of Se by carrots and broccoli in two growing seasons; (2) determine Se speciation in broccoli florets and carrots in two growing seasons; (3) determine the short-term transformation of organic Se into inorganic forms of Se in soils amended with Se-enriched *S. pinnata*; and (4) identify the microbiological community biomass and structure in soils amended with Se-enriched *S. pinnata* at postharvest of carrots and broccoli in one growing season.

## Materials and Methods

Selenium-enriched Prince’s Plume (*S. pinnata*) Pursh (Britton) was initially incorporated as an organic source of Se into the soil at different rates 3 years prior to onset of this 2-year study, as described by Bañuelos et al. (2015). The soil used in this 2-year field experiment was a Hanford sandy loam-coarse-loamy, mixed, thermic Typic Durixeralfs that had the following general properties; bulk density of 1.4 g cm^-3^, 55% sand, 40% silt, 5% clay, organic matter content of 7.4 kg^-1^, and a cation exchange capacity of 6.8 cmol_c_ kg^-1^. The Se-amended soil was contained within field-installed lysimeters, which had a 46 cm diameter, 152 cm length (7.5 cm above-ground), and were buried 1.1 m below the soil surface. The individual field-lysimeters (designated as microplot in text) were spaced 1.67 m apart (from center to center) with 3.65 m distance between rows. The added dried plant material had been milled to 100–300 μm particle size and initially contained ≈700 μg Se g^-1^ DW. In the plant tissue of *S. pinnata*, Bañuelos et al. (2015) reported that the predominate forms of extractable Se were ~86% organic (SeCyst), ~2% SeMet, 6% inorganic Se (selenite), and smaller percentages (<6%) of unidentifiable forms of Se compounds. As a check, we extracted *S. pinnata* plant tissue with water (e.g., mixed at ratio of 1:5 and incubated for 3 h), and found that the Se concentration in the water solution was as reported earlier by Bañuelos et al. (2015).

Based upon the study reported by Bañuelos et al. (2015), the amounts of absolute Se initially added 3 years prior to planting one (1st year) of the present study as Se-enriched *S. pinnata* plant material to each microplot as follows for each application rate (treatment) with six replications, respectively, (calculated as mg Se m^-2^); T0 (0 mg Se), T1 (105.4 mg Se), T2 (210.7 mg Se), T3 (421.4 mg Se), and T4 (842.9 mg Se). For this 2-year study, fertilizer (55 kg N ha^-1^, as 16-16-16) was applied prior to each planting as NH_4_SO_4_, P_2_O_5_, and K_2_O. Two soil samples were taken at 0–30 cm depth prior to planting one (1st year) and planting two (2nd year) and again at postharvest of planting two (0–120 cm) from each of the replicates for each *S. pinnata* treatment. A composite soil sample was made from each replicate for each treatment, prepared and analyzed for total and extractable Se, as described in Bañuelos et al. (2013), and other elements (water soluble and total) were analyzed with the inductively coupled spectrophotometer OES (Varian Vista-Pro, Santa Cruz, CA, USA). All Se samples were analyzed by an inductively coupled plasma mass spectrometer (Agilent 7500cx, Santa Clara, CA, USA). For Se speciation in soils, selenium speciation analyses were performed on fresh soil samples collected in triplicate, composited and repeated for six replicates from 0–30, 30–60, 60–90, to 90–120 cm from only the highest application rate (T4) at postharvest in planting two for both carrot and broccoli-planted soils. They were stored at -80°C and thawed prior to processing by adding deionized (DI) water to 100 g of fresh soil and mixing until a saturated soil paste was made (e.g., 1–2 mm of standing water visible on soil surface). The samples were then refrigerated for 24 h at 2°C, after which they were vacuum filtered thru Whatman #1 filter paper into 50 mL conical polypropylene tubes and frozen at -80°C for storage. Soil extracts were concentrated to 2 mL volume using a Labconco Speedvac concentrator, filtered thru a 0.22 μm Millipore syringe filters into 2.0 mL HPLC vials, and Se speciation analysis was performed by SAX-HPLC-ICP-MS (described later).

At planting one, broccoli (*B. oleracea L.* var. Marathon) and carrots (*Daucus carota* var. Sugarsnax 54) were planted in groups of three and nine, respectively, as 14 days old transplants into the already amended organic Se-treated soils within each microplot. At planting two, broccoli was similarly planted in soil previously planted with carrots in planting one and carrots were similarly planted in soil previously planted with broccoli in planting one. This type of planting strategy was intended to minimize soil related insect infestation and plant disease in both carrots and broccoli in planting two. For each planting, the application rates (treatments) of applied *S. pinnata* were randomly replicated six times in a complete randomized block design for carrots and broccoli in plantings one and two. Surface drip irrigation was set up in each microplot. The microplots received good quality water [electrical conductivity (EC) < 0.3 dS m^-1^] based on rate of evapotranspiration (ET) losses recorded by the California Irrigation Management Information System (CIMIS) located 2 km away. In addition, we evaluated a short-term (15 days) release and transformation of organic Se in soils amended with Se-enriched *S. pinnata*. For this study, soils in six unplanted microplots were freshly amended with *S. pinnata* that provided a total of 842.9 mg m^-2^ Se (based on T4 application rate) to a depth of 0–15 cm at a 70% soil field capacity. Three soil samples were collected from 0 to 15 cm depth at day 1, 7, and 15, respectively, from each of the six unplanted microplots and stored at -80°C until subsequent analysis for total and extractable Se, as well as organic forms of Se (described below).

### Tissue Harvest, Sample Preparation and Storage

After 121 and 137 days of growth, broccoli was harvested at planting one and two, respectively, at soil surface from each replicate, thoroughly rinsed with deionized water, and separated into florets, stems, and leaves. Total fresh weights were recorded for leaf, floret, and stem, and after florets were bulked from all three broccoli plants within each respective replicate; a total of six for each treatment. Each bulked sample was evenly divided into two parts. One part was lyophilized with a Labconco Freezone 2.5 freeze dryer (Labconco Corp., Kansas City, MS, USA) and stored at -80°C until speciation was performed (described later), while the other part of the bulked sample was dried at 65°C for 3 days. Carrots were harvested 133 and 165 days for planting one and two, respectively, and the nine plants were bulked together from each respective replicate (a total of six). Fresh weights were recorded for all bulked carrots and shoot samples from each replicate. Each bulked sample was further processed and scrubbed/rinsed thoroughly with deionized water to remove all soil particles from root and shoot. All oven dried plant samples from broccoli and carrots were ground to a fine powder in a UDY Cyclone mill equipped with a 1 mm mesh screen and further processed for Se analyses as described below. Freeze-dried samples of broccoli and carrots were processed and analyzed for different species of Se after aqueous extraction, as described later.

### ICP-MS of Total Se Concentrations and Se Speciation

Total Se was measured in floret and carrot samples using 500 mg dried ground plant material from each replicate, digested with HNO_3_, H_2_O_2_, and HCl, and analyzed by an inductively coupled plasma mass spectrometer (Agilent 7500 cx, Santa Clara, CA, USA). The National Institute of Standards and Technology (NIST) Wheat Flour (SRM 1567) was used as the standardized quality control for plant samples. The Se recovery rates were over 94% for the wheat flour standard, which has a concentration of 1.1 ± 0.2 μg Se g^-1^ DW, while the method detection limit was 50 ng Se g^-1^ DW. Other elements were analyzed from the plant digestate with the inductively coupled plasma spectrometer OES (Varian Vista-Pro, Santa Clara, CA, USA).

Analysis of soluble chemical forms of Se in aqueous proteolytic and non-proteolytic extracts were determined in broccoli and carrot samples that were freeze-dried and stored at -80°C by an Agilent 1200 HPLC equipped with a Hamilton PRP-X100 strong anion exchange column coupled to the Agilent 7500 CX ICP-MS (SAX-HPLC-ICP-MS). Selenium extraction efficiency from freeze-dried samples was ~61%. Details are described in great detail by Bañuelos et al. (2012).

### Soil PLFA Analysis

To identify the microbial communities in soils amended 3 years ago with *S. pinnata*, soil samples were collected from 0–5 to 5–30 cm (*n* = 6, respectively) at postharvest of broccoli and carrots from planting one of both the high treatment (T4) and control soils (T0) for phospholipid fatty acid (PLFA) analyses. PLFA were extracted from 5 g soil samples using a modified Bligh-Dyer methodology (Buyer et al., 2010). Lipids were directly extracted from soil samples using a mixture of chloroform: methanol: phosphate buffer (1:2:0.8). Phospholipids were separated from neutral lipids and glycolipids in a solid phase extraction column. After mild alkaline methanolysis, PLFA samples were qualitatively and quantitatively analyzed using an Agilent 6890 gas chromatograph (Agilent Technologies, Palo Alto, CA, USA) and fatty acids were identified using the MIDI PLFAD1 calibration mix and naming Table 2.5 (Buyer and Sasser, 2012; MIDI Inc., Newark, NJ, USA). Individual PLFA signatures were used to quantify the abundances of specific microbial groups in the collected soil samples (Buyer and Sasser, 2012). Gram + bacteria were identified with monounsaturated fatty acids and cyclopropyl 17:0 and 19:0, and eubacteria with 15:0, 17:0 cyclo, 15:1 iso, and 17:1 iso and 17:1 anteso. Fungi were identified and quantified with 18:2 ω6c, arbuscular mycrorrhizal fungi (AMF) with 16:1 ω5c, and actinomycetes with 10-methyl fatty acids (Frostegard et al., 1993; Zelles et al., 1994, 1995; Cavigelli et al., 1995; Blackwood and Buyer, 2004). Fatty acids were summed into biomarker groups according to Buyer and Sasser (2012).

### Statistical Analysis

Statistical analysis on relationships between plant and preplant soil Se concentrations was performed using Sigma Plot 13 (SSI, USA). One-way ANOVA with multiple comparisons using Duncan’s Method was utilized to compare significance among treatments (amounts of Se previously added to soils for both carrots and broccoli, respectively). Significance levels were expressed at both *P* < 0.05 and *P* < 0.001 levels.

For microbial analyses, statistical analysis was performed using SAS 9.3 (SAS Institute Inc., 2003). Two-way analysis of variance (ANOVA) was used followed by means separation with Student-Newman–Keuls (SNK) method to examine the significant differences among microbial community structure and biomass within each soil sampling site. A multivariate method (canonical analysis) was used to compare soil microbial communities in soils supporting broccoli and carrots with or without Se-enriched *S. pinnata*. In this analysis, MANOVA on the relative area of each biomarker was used to identify the linear combination of variables (referred to as canonical varieties) that best separated the soil microbial community structure at different sites. The canonical varieties were graphed to summarize group differences (Seber, 1984; Buyer et al., 1999, 2002). Significance levels were expressed at *P* < 0.05 level.

Note: Mention of trade names or commercial products in this publication is solely for the purpose of providing specific information and does not imply recommendation or endorsement by the U.S. Department of Agriculture. USDA is an equal opportunity provider and employer.

## Results

### Fate of *S. pinnata* Amendment in the Soil

Data show lower extractable Se concentrations in soil at preplant of planting two compared to planting one for broccoli and carrots. Mean total and extractable soil Se concentrations were measured as high as 1.65 mg kg^-1^ and 88 μg L^-1^, respectively, at preplant from 0 to 30 cm in planting one and as high as 0.95 mg kg^-1^ and 48.6 μg L^-1^ at preplant in planting two (see Supplementary Tables S1 and S2). Macro- and microelement concentrations are also shown from 0 to 30 cm in the soil at preplant between broccoli and carrots for both planting one and two, respectively (see Supplementary Tables S1 and S2). Analysis of soil samples collected at postharvest of planting two from T3 and T4 (highest application rates) showed that water soluble and total Se (and other elements) were present at different concentrations from 0 to 120 cm (See Supplementary Tables S3 and S4). Most soluble soil Se was present from 0 to 30 cm (see Supplementary Figure S1). Selenate and selenite were the two predominate species of Se present from 0 to 30 cm, while at the deeper depths (>30 cm) selenite, selenate, SeCys_2_, or SeCyst (1–10%) and methylselenocysteine (MeSeCys; 1–10%) were also detected (see Supplementary Figure S2). Evidence on the rapid transformation of organic Se (applied as Se-enriched *S. pinnata* to unplanted soil) to extractable forms of inorganic Se, e.g., selenite, was observed during the 15 days on our additional short term microplot study conducted on unplanted tiles amended with *S. pinnata* (see Supplementary Table S5).

### Effects on Broccoli and Carrot Yields

None of the plants exhibited any visible symptoms of abiotic stress, Se toxicity, or nutrient deficiency during the course of the 2-year study. Stand establishment for both carrots and broccoli was highly successful in both plantings one and two, irrespective of the previous *S. pinnata* treatments to the soil. Overall, there was no significant decrease in total fresh weight yield for either broccoli or carrots grown in soils amended with different *S. pinnata* treatments compared to control (T0) (**Tables 1** and **2**) for plantings one and two. There were, however, decreases in total broccoli yields between planting one and planting two, irrespective of treatment (**Tables 1** and **2**). This significant observation was likely the result of the later establishment of broccoli at planting two (4 weeks later compared to planting one); broccoli in planting two experienced additional stress with the warmer temperatures (data not presented).

**Table 1:** Total fresh weight yields of broccoli grown in soil amended with *S. pinnata* for planting one and two.

	Fresh weight^†^			
Treatment^‡^ #	Leaf	Floret	Stem	Total
		(g microplot^-1^)	
**Planting one:**				
T0	520^ab^	309^a#^	255^a^	1083^a^
	(80)	(82)	(49)	(153)
T1	563^ab^	335^a^	279^a^	1177^a^
	(94)	(42)	(51)	(163)
T2	438^b^	343^a^	256^a^	1037^a^
	(105)	(66)	(44)	(154)
T3	560^ab^	381^a^	277^a^	1218^a^
	(78)	(47)	(19)	(105)
T4	646^a^	382^a^	303^a^	1019^a^
	(238)	(73)	(50)	(301)
**Planting two:**			
T0	224^a^	126^b^	160^a^	492^b^
	(59)	(54)	(53)	(153)
T1	205^a^	149^ab^	188^a^	542^ab^
	(31)	(66)	(40)	(86)
T2	284^a^	170^ab^	251^a^	527^ab^
	(118)	(82)	(113)	(257)
T3	281^a^	199^ab^	186^a^	636^ab^
	(120)	(59)	(70)	(245)
T4	278^a^	211^a^	235^a^	725^a^
	(71)	(23)	(40)	(132)

*^†^Each value represents the mean of six replicates followed by the standard deviation in parenthesis.*

*^‡^Treatments correspond to amounts of Se added via *S. pinnata* (mg Se m^-2^): T0(0), T1(105.4), T2(210.7), T3(421.4), and T4(842.9).*

*^*#*^Different letters within each column represent a significant difference at the *P* < 0.05 level.*

**Table 2:** Total fresh weight yields of carrots grown in soil amended with *S. pinnata* for planting one and two.

	Fresh weight^†^		
Treatment^‡^ #	Carrot	Shoot	Total
		(g microplot^-1^)	
**Planting one:**			
T0	758^a#^	318^a^	1076^a^
	(184)	(88)	(271)
T1	753^a^	323^a^	1076^a^
	(108)	(78)	(177)
T2	714^a^	329^a^	1043^a^
	(114)	(80)	(166)
T3	685^a^	294^a^	979^a^
	(115)	(62)	(168)
T4	800^a^	316^a^	1116^a^
	(146)	(89)	(203)
**Planting two:**			
T0	669^a^	254^a^	923^a^
	(138)	(21)	(158)
T1	587^a^	258^a^	845^a^
	(117)	(64)	(148)
T2	784^a^	322a	1106^a^
	(183)	(55)	(230)
T3	646^a^	300^a^	946^a^
	(197)	(64)	(249)
T4	655^a^	307^a^	962^a^
	(65)	(65)	(241)

*^†^Each value represents the mean of six replicates followed by the standard deviation in parenthesis.*

*^‡^Treatments correspond to amounts of Se added via *S. pinnata* (mg Se m^-2^): T0(0), T1(105.4), T2(210.7), T3(421.4), and T4(842.9).*

*^*#*^Different letters within each column represent a significant difference at the *P* < 0.05 level.*

### Effects on Total Plant Se Accumulation

**FIGURE 1:**
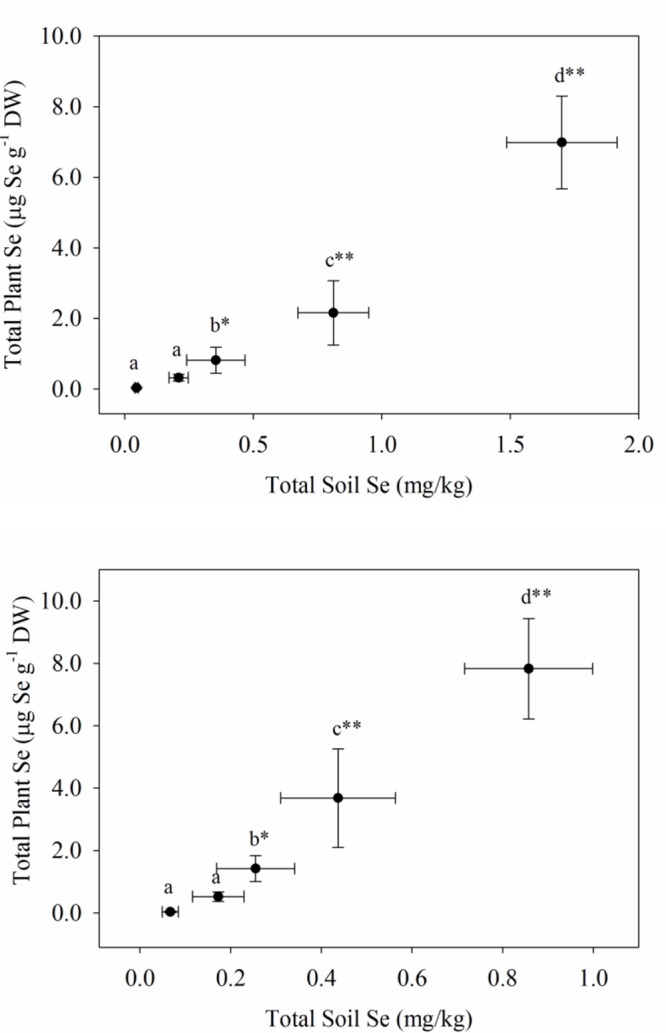
**Total Se concentrations in broccoli florets grown in soil amended with Se at different rates of *S. pinnata* for planting one and two.** Each value represents the mean of six replicates with standard deviation bars for both soil (*x*-axis) and plants (*y*-axis). Different letters within each column represent a significant difference at the *P < 0.05, **P < 0.001 level. Treatments correspond to amounts of Se added via *S. pinnata* (mg Se m^-2^): T0(0), T1(105.4), T2(210.7), T3(421.4), and T4(842.9).

**FIGURE 2:**
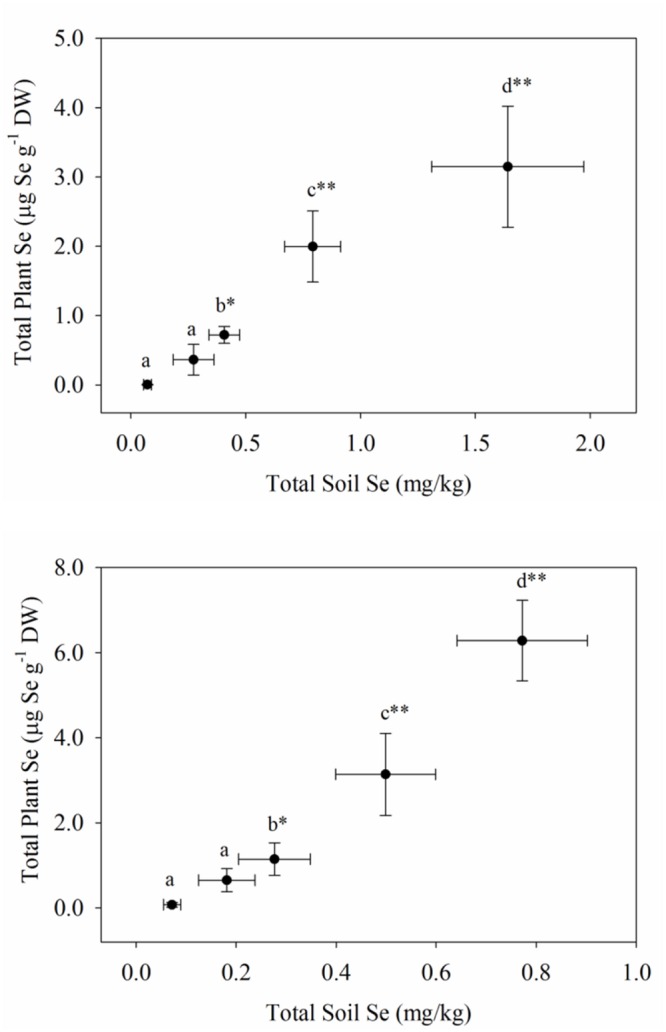
**Total Se concentrations in carrots grown in soil amended with Se at different rates of *S. pinnata* for planting one and two.** Each value represents the mean of six replicates with standard deviation bars for both soil (*x*-axis) and plants (*y*-axis). Different letters within each column represent a significant difference at the *P < 0.05, **P < 0.001 level. Treatments correspond to amounts of Se applied via *S. pinnata* added (mg Se m^-2^): T0(0), T1(105.4), T2(210.7), T3(421.4), and T4(842.9).

The uptake of Se readily occurred in both plantings of carrots and broccoli, although the Se-enriched *S. pinnata* was initially applied to the soil 3 and 4 years ago for plantings one and two, respectively. In our study, selenium concentrations in the broccoli floret increased from 0.03 (T0, control) to a high of 6.99 mg kg^-1^ (T4) and from 0.04 (T0, control) to a high of 7.83 mg kg^-1^ (T4) for planting one and two, respectively (**Figure 1**), while Se concentrations in carrot increased from 0.01 (T0, control) to a high of 3.15 mg kg^-1^ (T4) and from 0.05 (T0, control) to an inexplicably high of 6.28 mg kg^-1^ (T4) for planting one and two, respectively (**Figure 2**). Selenium uptake efficiency was considerably improved by carrots in planting two. There were robust relationships (due to inhomogeneous variances) between the measured preplant soil Se concentrations and total Se levels in broccoli florets and carrot for planting one and two, respectively. We did not ascertain whether organic Se added three to 4 years ago as *S. pinnata* was absorbed as organic Se, inorganic Se, or as both in the present study. In addition to plant Se concentrations, Supplementary Tables S6 and S7 show the macro- and micronutrient concentrations in both broccoli florets and carrots for both planting one and two.

We only performed Se speciation analyses in aqueous extracts from carrots and broccoli at the highest amendment rate (T4), due to the earlier reported observations that Se speciation did not significantly vary in either broccoli and carrots growing in soils amended with different rates of *S. pinnata* (Bañuelos et al., 2015). In broccoli, we identified an average of 50, 11, and 5% as SeMet, SeCys_2_, and MeSeCys, respectively, for both planting one and two (**Tables 3** and **4**), while carrots exhibited 65, 11, and 12% as SeMet, SeCys_2_, and MeSeCys, respectively, in planting one and two.

**Table 3:** Percentage of soluble Se species in broccoli florets and carrot roots grown in soil amended with *S. pinnata* at highest rate (T4) for planting one.

Crop Extracted	SeCys_2_ (%)	MeSeCys (%)	Selenite (%)	SeMet (%)	Selenate (%)	GSSeSG (%)	Unknown (%)
Broccoli	15.0^†^	5.0	0	58.5	0	17.0	4.5
	(1.6)	(2.6)	0	(9.2)	0	(3.0)	(1.2)
Carrots	12.0	6.0	0	78.5	2.5	0	2.0
	(4.5)	(5.7)	0	(10.3)	(0.5)	0	(0.8)

*^†^Values represent the mean percentage of Se compounds detected in aqueous extracts of protease XIV digested samples (*n* = 6) with standard deviation (SD) in parenthesis.*

**Table 4:** Percentage of soluble Se species in broccoli florets and carrot roots grown in soil amended with *S. pinnata* at highest rate (T4) for planting two.

Crop Extracted	SeCys_2_ (%)	MeSeCys (%)	Selenite (%)	SeMet (%)	Selenate (%)	GSSeSG (%)	Unknown (%)
Broccoli	6.0^†^	4.1	0	37.5	0	34	18.5
	(1.5)	(1.5)	0	(7.5)	0	(10.7)	(2.5)
Carrots	10.9	17.3	0	56.3	4.5	0	11
	(2.5)	(5.4)	0	(9.3)	(1.1)	0	(3.1)

*^†^Values represent the mean percentage of Se compounds detected in aqueous extracts of protease XIV digested samples (*n* = 6) with standard deviation (SD) in parenthesis.*

### Effects on Total Amount and Biomarker Values of PLFA in *S. pinnata* Amended Soil Growing Carrots and Broccoli

**FIGURE 3:**
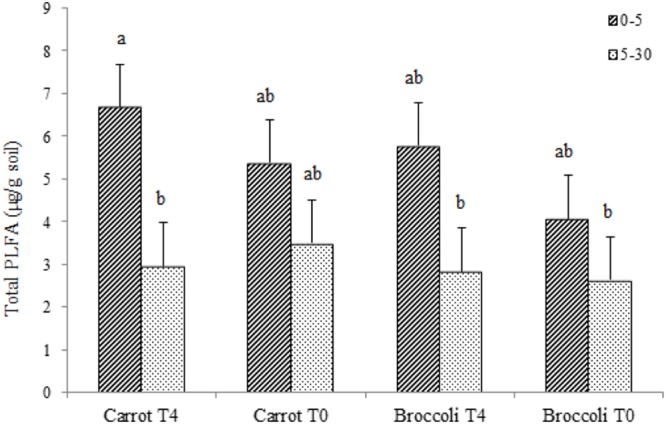
**Total phospholipid fatty acids (PLFAs) in Se amended soils grown with (carrot T4 and broccoli T4) and without (carrot T0 and broccoli T0) at 0–15 and 5–30 cm depths.** Different letters represent a significant difference at the *P* < 0.05 level for each respective depth. Values represent the mean (*n* = 6) and standard deviation bar.

**FIGURE 4:**
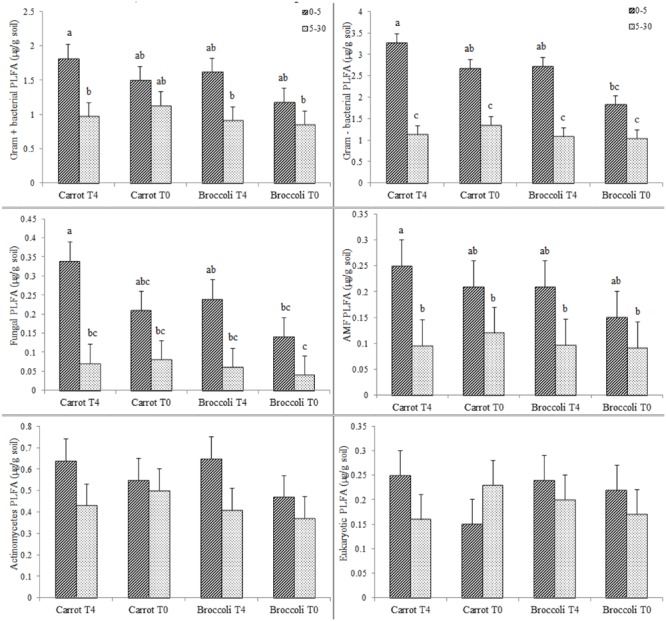
**Gram+, Gram-, Fungal, AMF, Actinomycetes, and Eukaryotic PLFAs in Se amended soil grown with (carrot T4 and broccoli T4) and without (carrot T0 and broccoli T0) at 0–5 and 5–30 cm depths.** Different letters represented a significant difference at the *p* < 0.05 level for each respective depth. Values represent the mean (*n* = 6) and standard deviation bar.

We performed an initial identification on the microbial communities existing at postharvest of planting one in both control (T0) and *S. pinnata* amended soils at the highest amendment rate (T4). Total PLFA showed little significant differences for carrot and broccoli soils at 0–5 and 5–30 cm that were amended with T4 compared to control (T0) at postharvest of planting one (**Figure 3**). Phospholipid fatty acid analyses at postharvest of planting one showed that the composition of total microbial biomass, AMF, actinomycetes, Gram + bacterial PLFA was similar between high amendment rate (T4) and unamended soil (T0, control) at both 0–5 and 5–30 cm, respectively, in both broccoli and carrot soils (**Figure 4**), although PLFA values were overall greater from 0 to 5 cm compared to 5–30 cm, irrespective of treatment.

Canonical multivariate analyses are shown for both sampling depths of 0–5 and 5–30 cm from unamended soil (control,T0) and high amendment rate (T4) for both broccoli and carrots at postharvest of planting one (see Supplementary Figures S3 and S4). The soil microbial communities were similar at 0–5 cm for both broccoli and carrots, irrespective of treatment, but they were significantly different (*P* < 0.05) when comparing 0–5 cm for both carrots and broccoli, irrespective of treatment. In carrot planted soils (0–5 cm), the discriminate analysis showed that higher fungi and AMF values were observed in unamended soil (control, T0) compared to high amendment rate (T4), and higher Gram – bacteria were observed in T4 compared to control (T0) soil (0–5 cm). Actinomycetes populations remained similar for both treatments (T4 and T0) in broccoli and carrot planted soils (0–5 or 5–30 cm). Overall, *S. pinnata* applied at the high amended rate (T4) had no significant effect on the amount and biomarker values of PLFA, microbial composition, and microbial structure in (see Supplementary Table S8). Hence, microbial analyses in the soil were only performed once at postharvest of planting one.

## Discussion

Successfully producing Se-biofortified carrots and broccoli 3 and 4 years later after initially applying organic Se as Se-enriched *S. pinnata* to the soil, clearly demonstrates the unique bi-functionality of *S. pinnata* both as an effective Se phytoremediation tool (Feist and Parker, 2001; Freeman and Bañuelos, 2011) and as a continued source of organic-Se for growing Se-biofortified carrots and broccoli. Importantly, the one-time application of organic Se as Se-enriched *S. pinnata* resulted in different preplant soil Se concentrations between planting one and two. The lower preplant soil Se concentrations at planting two did not result in lower plant Se concentrations in carrot and broccoli. In contrast, they were even higher in both crops grown at all total soil Se concentrations associated with each application rate. In fact, the clear differences observed in tissue Se concentrations between broccoli and carrot in planting one inexplicably disappeared in planting two; there was no clear difference in the accumulation of Se between broccoli and carrots. Compared to the organic species of Se present in the soil, it is possible that more inorganic Se was present in the soil compared to planting one and subsequently more Se was taken up by both carrots and broccoli in planting two (see below). In addition, higher transpiration rates (not measured) associated with warmer temperatures and the later and longer growing seasons in planting two may have also contributed to the higher plant Se concentrations for both carrots and broccoli in planting two. The predominant presence of inorganic species of Se, e.g., selenite, selenate, in the soil clearly indicates that organic forms of Se originating from the Se-enriched *S. pinnata* applied to the soil over 4 years ago, had been converted to inorganic Se within the soil, mostly (>90%) as selenite and then selenate. The presence of soil Se 3 and 4 years later at preplant of planting one and two indicates that organic Se applied via *S. pinnata* did not need to be reapplied for this current study. The lower extractable soil Se concentrations measured at preplant of planting two suggests that extractable Se was both absorbed by the plants and/or lost by leaching during planting one. The measurement of Se to a depth of 120 cm at postharvest of planting two clearly shows that Se applied via *S. pinnata* is partially converted to mobile forms of inorganic Se. Selenium’s continued presence in the soil at preplant of planting one and two may also be a result of California’s extreme drought conditions and lack of precipitation for the last 4 years; hence, significant leaching was likely minimized in this light-textured soil. Additionally, we assume that untransformed selenoamino acids contained within the plant matrix of *S. pinnata*, e.g., SeCyst, added to soil 4 years ago can remain as a reserve of organic Se in the amended soil and will quickly transform into inorganic Se under ideal soil moisture conditions [as observed in our 15 days trial with *S. pinnata* amended (T4) soils]. The rapid and easy extraction of Se from wetted *S. pinnata* (described in Materials and Methods) and the dissolution of organic Se into inorganic forms of Se, indicates that soluble Se will be quickly available for plant uptake in moist soils amended with *S. pinnata.* Other studies have reported that plants absorb Se more rapidly from organic sources of Se compared to inorganic forms of Se (Kikkert and Berkelaar, 2013). In addition, broccoli as a member of the *Brassica* genus (known for its affinity for sulfur) can readily absorb Se, due to the chemical and physical similarly between Se and S (Bañuelos, 2002). The fibrous root system and larger root surface area of broccoli, should allow this crop to access more Se than carrots with a taproot and fine roots. Surprisingly, carrot Se concentrations were almost as high as broccoli in planting two, even though the carrot is a non-*Brassica* species. This inexplicable observation may be a result of the carrot root making direct contact with inorganic Se in the soil amended with Se-enriched *S. pinnata*.

In the carrot and broccoli, percentages of the speciated forms of Se were similar to those reported for broccoli by Bañuelos et al. (2015) but the percentage of SeMet detected (65%) in carrots was significantly greater than 33%, as reported by Bañuelos et al. (2015). This unexpected difference between data reported by Bañuelos et al. (2015) and our present study suggest that the detection sensitivity of Se species may vary in carrot tissue depending on extraction technique. In Bañuelos et al. (2015), aqueous extractions of soluble Se were obtained from fresh samples of carrot frozen at -80°C, while aqueous extractions of soluble Se were obtained from freeze-dried samples of broccoli and carrot stored at -80°C in this study. Presumably, concentrations of proteolytic extractable Se from carrots should be greater from a freeze-dried sample from which water has been removed by the freeze-drying process and the content of the Se species, e.g., SeMet, were more concentrated compared to fresh samples stored at -80°C. Preliminary investigation by our laboratory confirmed that lower amounts of extractable Se were detected from carrots fresh frozen and stored at -80°C compared to freeze-dried and stored at -80°C.

We did not investigate the possibility of whether the application of inorganic Se would influence the Se speciation in the carrot and broccoli differently than the organic application of Se as Se-enriched *S. pinnata*, as suggested by Poblaciones et al. (2014). The speciation of Se within a plant species will also vary and depend upon the crop species, as observed with broccoli and carrots but in this study. SeMet was the predominate selenoamino acid in carrots [as reported by Kapolna et al. (2009)] and broccoli after amending soil 3–4 years later with *S. pinnata*. The identification of MeSeCys in both broccoli and carrots (albeit at surprisingly low percentages) is, however, of great interest for future studies, as MeSeCys is purposed to confer additional anticarcinogenic properties (Zhu et al., 2009), if this form of Se is stored more effectively in the body (Rayman, 2008). In any case, additional research is needed to explore Se speciation in different crops biofortified with different sources of inorganic and organic Se or different forms of foliarly applied Se.

There was no significant effect on the microbial community in soil amended with *S. pinnata* at postharvest of planting one compared to unamended soil 3 years. Similarly, Chander and Joergensen (2007) reported that high Se concentrations, e.g., 20 μg g^-1^, had no effect on other soil microbial indices, e.g., microbial biomass C and N, ATP, AEC, ATP-to-microbial biomass C, and qCO2. In this study, we were unable to ascertain whether the microbial community varied in the soil during the growing season (activity not performed), since we only evaluated the relationship between Se-enriched *S. pinnata* added to the soil 3 years ago and the microbial community present at postharvest of planting one. To determine if this source of organic Se as *S. pinnata* directly affects the microbial community, future studies should include the evaluation of the microbial community during the crop’s active growing season and in soils freshly amended with Se-enriched *S. pinnata*.

Our study shows that one-time application of Se-enriched *S. pinnata* at tested rates (e.g., T3 and T4) still provided sufficient Se to the soil to significantly biofortify carrots and broccoli with Se 3 and 4 years later. On a field-scale application basis, applying inorganic Se fertilizers to Se-deficient soils may be more practical in many countries because of the accessibility of inorganic fertilizers compared to organic sources of Se as *S. pinnata*. In Se rich soils, e.g., Enshi, China, regions of California and Colorado, USA, *S. pinnata* can be easily grown as a potential organic source of Se, however, not all Se-deficient soils are located near Se-rich soils throughout the world. In addition, application strategies would need to be developed for growers attempting to apply large quantities of *S. pinnata* on a hectare basis for Se-deficient soil. For example, based on T3 rate of application and Se concentrations measured in *S. pinnata*, 6097 kg of *S. pinnata*/hectare would need to be applied to biofortify carrots and broccoli under similar growing conditions, as illustrated in the 2-year current study. The continued availability of Se for plant uptake 4 years after initial application of *S. pinnata* to soil certainly warrants future research on studying the continued availability of Se that may exceed 4 years. Importantly, additional studies should compare the economics related to multiple applications of inorganic Se as a fertilizer compared to one time application of organic Se as *S. pinnata*, e.g., every 4 years (or even more) to Se-deficient soils with *S. pinnata*. Lastly, it would also be helpful for determining future plant Se availability for continued biofortification, if future studies included performing mass-balance calculations on Se lost from the soil by plant uptake, leaching, and volatilization.

## Author Contributions

GB designed and wrote the majority of the paper. IA executed and supervised the field study, as well as performed all Se analysis. SD performed all activities related to PLFA analyses. MZ performed Se speciation microplot study in unplanted soils amended with *S. pinnata*.

## Conflict of Interest Statement

The authors declare that the research was conducted in the absence of any commercial or financial relationships that could be construed as a potential conflict of interest. The reviewer TS and handling Editor declared their shared affiliation, and the handling Editor states that the process nevertheless met the standards of a fair and objective review.
