# Supplementary material for: Continued Selenium Biofortification of Carrots and Broccoli Grown in Soils Once Amended with Se-enriched S. pinnata
Source: Front Plant Sci. 2016 Aug 23;7:1251. doi: 10.3389/fpls.2016.01251 (PMC4993952; doi:10.3389/fpls.2016.01251)
Supplement: Supplementary file 1 [file Table_1.DOCX]

Supplementary Material

**Continued selenium biofortification of carrots and broccoli grown in soils amended with Se-enriched *S. pinnata***

Bañuelos, G.S.*, Arroyo, I., Dangi, S.R., Zambrano, M.C.

*** Correspondence:** Corresponding Author: gary.banuelos@ars.usda.gov

# Supplementary Tables

## Supplementary Figures

**Supplementary Table 1. Mean water soluble and total elemental concentrations in soils amended with *S. pinnata* (0-30 cm) at preplant of planting one for carrots and broccoli^†^.**

|  |  |  | Water soluble | | | | | | | | | | | | |
| --- | --- | --- | --- | --- | --- | --- | --- | --- | --- | --- | --- | --- | --- | --- | --- |
|  |  | EC | B | Ca | Cu | Fe | K | Mg | Mn | Mo | Na | P | S | Zn | Se |
| Treatment^‡^ | pH | mS/cm | - - - - - - - - - - - - - - - - - - -- - - - - -- - - - - - - - - mg L^-1^ - - - - --- - - -- - - - - - - - - - - - - - - - - - - - - - - | | | | | | | | | | | | µg L^-1^ |
| T0 | 7.89 | 0.52 | 0.07 | 71 | 0.03 | 0.06 | 14 | 15 | 0.06 | 0.01 | 13 | 1.5 | 11 | 0.0 | 1 |
|  | *0.16* | *0.13* | *0.03* | *17* | *0.01* | *0.03* | *5* | *4* | *0.06* | *0.00* | *4* | *0.7* | *3* | *0.0* | *0* |
|  |  |  |  |  |  |  |  |  |  |  |  |  |  |  |  |
| T1 | 7.81 | 0.6 | 0.08 | 82 | 0.03 | 0.06 | 16 | 18 | 0.09 | 0.01 | 15 | 1.8 | 14 | 0.0 | 10 |
|  | *0.19* | *0.2* | *0.02* | *29* | *0.01* | *0.04* | *5* | *7* | *0.07* | *0.00* | *4* | *0.9* | *5* | *0.0* | *3* |
|  |  |  |  |  |  |  |  |  |  |  |  |  |  |  |  |
| T2 | 7.84 | 0.62 | 0.1 | 83 | 0.03 | 0.05 | 17 | 18 | 0.07 | 0.01 | 16 | 1.7 | 15 | 0.0 | 19 |
|  | *0.15* | *0.18* | *0.04* | *23* | *0.01* | *0.04* | *9* | *6* | *0.09* | *0.00* | *3* | *0.9* | *4* | *0.0* | *5* |
|  |  |  |  |  |  |  |  |  |  |  |  |  |  |  |  |
| T3 | 7.86 | 0.57 | 0.08 | 79 | 0.03 | 0.06 | 18 | 17 | 0.13 | 0.01 | 15 | 1.6 | 13 | 0.0 | 42 |
|  | *0.15* | *0.24* | *0.01* | *33* | *0.01* | *0.04* | *6* | *9* | *0.15* | *0.00* | *6* | *0.8* | *6* | *0.0* | *12* |
|  |  |  |  |  |  |  |  |  |  |  |  |  |  |  |  |
| T4 | 7.84 | 0.56 | 0.1 | 76 | 0.03 | 0.09 | 18 | 16 | 0.13 | 0.02 | 13 | 2.1 | 12 | 0.0 | 88 |
|  | *0.15* | *0.15* | *0.02* | *21* | *0.01* | *0.09* | *5* | *5* | *0.12* | *0.00* | *4* | *1.2* | *3* | *0.0* | *15* |
|  |  |  |  |  |  |  |  |  |  |  |  |  |  |  |  |
|  |  |  | Total | | | | | | | | | | | | |
|  |  |  | B | Ca | Cu | Fe | K | Mg | Mn | Mo | Na | P | S | Zn | Se |
| Treatment^‡^ |  |  | - - - - - - - -- - - - - - - - - - - - - - - - - - - - -- - - - - mg kg^-1^ - - - - - - - - - -- - - - - - - - - - - - - - - - - - - - - - - - | | | | | | | | | | | | |
| T0 |  |  | 2.3 | 2907 | 12 | 16062 | 2579 | 3217 | 276 | 0.37 | 103 | 506 | 83 | 37 | 0.06 |
|  |  |  | *0.6* | *297* | *3* | *810* | *88* | *85* | *86* | *0.10* | *23* | *99* | *21* | *10* | *0.02* |
|  |  |  |  |  |  |  |  |  |  |  |  |  |  |  |  |
| T1 |  |  | 2.2 | 2836 | 12 | 16030 | 2592 | 3167 | 281 | 0.34 | 105 | 519 | 83 | 37 | 0.24 |
|  |  |  | *0.7* | *301* | *3* | *662* | *94* | *101* | *76* | *0.06* | *18* | *102* | *25* | *9* | *0.07* |
|  |  |  |  |  |  |  |  |  |  |  |  |  |  |  |  |
| T2 |  |  | 1.9 | 2908 | 12 | 16026 | 2566 | 3168 | 307 | 0.36 | 105 | 532 | 83 | 39 | 0.38 |
|  |  |  | *0.7* | *325* | *3* | *494* | *98* | *115* | *116* | *0.09* | *15* | *109* | *14* | *10* | *0.09* |
|  |  |  |  |  |  |  |  |  |  |  |  |  |  |  |  |
| T3 |  |  | 2.5 | 2892 | 11 | 16064 | 2595 | 3201 | 279 | 0.37 | 103 | 523 | 86 | 36 | 0.8 |
|  |  |  | *0.9* | *210* | *3* | *1005* | *76* | *76* | *83* | *0.09* | *20* | *129* | *20* | *8* | *0.12* |
|  |  |  |  |  |  |  |  |  |  |  |  |  |  |  |  |
| T4 |  |  | 2.3 | 2872 | 12 | 16008 | 2619 | 3176 | 313 | 0.38 | 102 | 525 | 89 | 38 | 1.65 |
|  |  |  | *0.8* | *265* | *3* | *798* | *54* | *90* | *116* | *0.06* | *20* | *103* | *11* | *10* | *0.31* |
| **^†^**Values are the means from twelve replicates with the standard deviation italicized. | | | | | | | | |  |  |  |  |  |  |  |
| ^‡^Treatments correspond to amounts of Se added via *S. pinnata* (mg Se m^-2^): T0(0), T1(105.4), T2(210.7), T3(421.4), and T4(842.9). | | | | | | | | | | | | | | | |

**Supplementary Table 2. Mean water soluble and total elemental concentrations in soils amended with *S. pinnata* (0-30 cm) at preplant of planting two for carrots and broccoli^†^.**

|  |  |  | Water Soluble | | | | | | | | | | | | |
| --- | --- | --- | --- | --- | --- | --- | --- | --- | --- | --- | --- | --- | --- | --- | --- |
|  |  | EC | B | Ca | Cu | Fe | K | Mg | Mn | Mo | Na | P | S | Zn | Se |
| Treatment^‡^ | pH | mS/cm | - - - - - - - - - - - - - - - - - - - - - - - - - - - - - - - - - - mg L^-1^ - - - - - - - - - - - - - - - - - - - - - - - - - - - - - - - - - | | | | | | | | | | | | µg L^-1^ |
| T0 | 7.66 | 0.30 | 0.06 | 30 | 0.03 | 0.53 | 6 | 6 | 0.13 | 0.04 | 28 | 0.8 | 9 | 0.0 | 1.6 |
|  | *0.22* | *0.05* | *0.02* | *5* | *0.01* | *0.36* | *2* | *1* | *0.09* | *0.02* | *5* | *0.7* | *3* | *0.0* | *0.4* |
|  |  |  |  |  |  |  |  |  |  |  |  |  |  |  |  |
| T1 | 7.66 | 0.30 | 0.07 | 30 | 0.02 | 0.46 | 6 | 7 | 0.24 | 0.05 | 28 | 0.8 | 10 | 0.0 | 5.2 |
|  | *0.19* | *0.07* | *0.03* | *7* | *0.01* | *0.27* | *3* | *2* | *0.25* | *0.02* | *6* | *0.5* | *5* | *0.0* | *2.0* |
|  |  |  |  |  |  |  |  |  |  |  |  |  |  |  |  |
| T2 | 7.59 | 0.38 | 0.08 | 36 | 0.03 | 0.40 | 6 | 8 | 0.15 | 0.04 | 36 | 1.0 | 16 | 0.0 | 11.1 |
|  | *0.26* | *0.17* | *0.03* | *12* | *0.01* | *0.37* | *2* | *3* | *0.10* | *0.02* | *17* | *0.6* | *13* | *0.0* | *3.2* |
|  |  |  |  |  |  |  |  |  |  |  |  |  |  |  |  |
| T3 | 7.56 | 0.43 | 0.08 | 44 | 0.02 | 0.40 | 7 | 9 | 0.17 | 0.04 | 36 | 0.7 | 18 | 0.0 | 23.7 |
|  | *0.18* | *0.18* | *0.03* | *21* | *0.01* | *0.38* | *3* | *5* | *0.17* | *0.02* | *16* | *0.6* | *11* | *0.0* | *6.2* |
|  |  |  |  |  |  |  |  |  |  |  |  |  |  |  |  |
| T4 | 7.53 | 0.42 | 0.10 | 44 | 0.03 | 0.43 | 8 | 9 | 0.31 | 0.04 | 37 | 1.1 | 18 | 0.0 | 48.6 |
|  | *0.23* | *0.15* | *0.03* | *16* | *0.00* | *0.27* | *3* | *4* | *0.30* | *0.01* | *14* | *0.6* | *12* | *0.0* | *17.5* |
|  |  |  |  |  |  |  |  |  |  |  |  |  |  |  |  |
|  |  |  | Total | | | | | | | | | | | | |
|  |  |  | B | Ca | Cu | Fe | K | Mg | Mn | Mo | Na | P | S | Zn | Se |
| Treatment^‡^ |  |  | - - - - - - - - - - - - - - - - - - - - - - - - - - - - - - - - - - - - - mg kg^-1^ - - - - - - - - - - - - - - - - - - - - - - - - - - - - - - - - - - - - | | | | | | | | | | | | |
| T0 |  |  | 1.7 | 2870 | 14 | 16351 | 2547 | 3251 | 279 | 0.6 | 144 | 500 | 74 | 41 | 0.09 |
|  |  |  | *0.8* | *147* | *3* | *927* | *113* | *164* | *33* | *0.6* | *12* | *74* | *14* | *8* | *0.07* |
|  |  |  |  |  |  |  |  |  |  |  |  |  |  |  |  |
| T1 |  |  | 1.8 | 2801 | 14 | 16304 | 2526 | 3214 | 275 | 0.8 | 147 | 479 | 73 | 39 | 0.17 |
|  |  |  | *1.1* | *160* | *2* | *271* | *87* | *112* | *31* | *0.5* | *9* | *35* | *16* | *6* | *0.06* |
|  |  |  |  |  |  |  |  |  |  |  |  |  |  |  |  |
| T2 |  |  | 1.8 | 2872 | 14 | 16213 | 2570 | 3266 | 277 | 0.5 | 154 | 477 | 78 | 41 | 0.27 |
|  |  |  | *1.1* | *161* | *2* | *409* | *65* | *85* | *33* | *0.3* | *27* | *58* | *18* | *6* | *0.08* |
|  |  |  |  |  |  |  |  |  |  |  |  |  |  |  |  |
| T3 |  |  | 2.0 | 2847 | 16 | 16922 | 2557 | 3268 | 284 | 0.6 | 157 | 486 | 83 | 40 | 0.49 |
|  |  |  | *1.0* | *101* | *6* | *2189* | *78* | *126* | *41* | *0.4* | *23* | *49* | *21* | *4* | *0.14* |
|  |  |  |  |  |  |  |  |  |  |  |  |  |  |  |  |
| T4 |  |  | 2.1 | 2775 | 13 | 15880 | 2535 | 3206 | 267 | 0.4 | 150 | 471 | 84 | 39 | 0.92 |
|  |  |  | *0.8* | *222* | *2* | *347* | *72* | *135* | *28* | *0.3* | *22* | *82* | *23* | *6* | *0.29* |
| **^†^**Values are the means from twelve replicates with the standard deviation italicized. | | | | | | | | |  |  |  |  |  |  |  |
| ^‡^Treatments correspond to amounts of Se added via *S. pinnata* (mg Se m^-2^): T0(0), T1(105.4), T2(210.7), T3(421.4), and T4(842.9). | | | | | | | | | | | | | | | |

**Supplementary Table 3. Mean water soluble concentrations in soils amended with *S. pinnata* at postharvest of planting two for carrots and broccoli^†^.**

|  |  | Water soluble | | | | | | | | | | | | | | |
| --- | --- | --- | --- | --- | --- | --- | --- | --- | --- | --- | --- | --- | --- | --- | --- | --- |
|  | Depth |  | EC | B | Ca | Cu | Fe | K | Mg | Mn | Mo | Na | P | S | Zn | Se |
| Treatment^‡^ | (cm)^¶^ | pH | mS/cm | - - - - - - - - - - - - - - - - - - - - - - - - - - - mg L^-1^ - - - - - - - - - - - - - - - - - - - - - - - - - - - - - | | | | | | | | | | | | µg L^-1^ |
| T0 | 0-30 | 7.82 | 0.28 | 0.14 | 28 | 0.02 | 0.64 | 5 | 6 | 0.06 | 0.02 | 25 | 0.93 | 10 | 0.01 | 1 |
|  |  | *0.14* | *0.02* | *0.11* | *4* | *0.01* | *0.34* | *2* | *1* | *0.04* | *0.01* | *4* | *0.47* | *2* | *0.01* | *0* |
|  |  |  |  |  |  |  |  |  |  |  |  |  |  |  |  |  |
| T1 | 0-30 | 7.77 | 0.27 | 0.13 | 25 | 0.02 | 0.61 | 5 | 5 | 0.06 | 0.02 | 25 | 0.92 | 10 | 0.03 | 4 |
|  |  | *0.14* | *0.05* | *0.13* | *6* | *0.01* | *0.4* | *2* | *2* | *0.04* | *0.01* | *5* | *0.34* | *4* | *0.05* | *1* |
|  |  |  |  |  |  |  |  |  |  |  |  |  |  |  |  |  |
| T2 | 0-30 | 7.84 | 0.28 | 0.17 | 28 | 0.02 | 0.43 | 5 | 6 | 0.06 | 0.02 | 26 | 0.81 | 11 | 0.03 | 7 |
|  |  | *0.13* | *0.05* | *0.12* | *6* | *0.01* | *0.23* | *3* | *2* | *0.06* | *0.01* | *6* | *0.35* | *4* | *0.07* | *2* |
|  |  |  |  |  |  |  |  |  |  |  |  |  |  |  |  |  |
| T3 | 0-30 | 7.78 | 0.26 | 0.11 | 25 | 0.02 | 0.76 | 5 | 5 | 0.06 | 0.02 | 24 | 0.77 | 9 | 0.03 | 14 |
|  |  | *0.16* | *0.03* | *0.1* | *5* | *0.01* | *0.37* | *3* | *1* | *0.05* | *0.01* | *3* | *0.42* | *2* | *0.07* | *3* |
|  |  |  |  |  |  |  |  |  |  |  |  |  |  |  |  |  |
|  | 30-60 | 7.8 | 0.2 | 0.13 | 17 | 0.01 | 0.73 | 3 | 4 | 0.02 | 0.02 | 21 | 0.55 | 6 | 0.08 | 4 |
|  |  | *0.16* | *0.02* | *0.11* | *3* | *0.01* | *0.37* | *1* | *1* | *0.02* | *0.02* | *4* | *0.25* | *2* | *0.13* | *1* |
|  |  |  |  |  |  |  |  |  |  |  |  |  |  |  |  |  |
|  | 60-90 | 7.76 | 0.18 | 0.14 | 13 | 0.01 | 0.7 | 3 | 3 | 0.02 | 0.02 | 21 | 0.75 | 5 | 0.01 | 3 |
|  |  | *0.16* | *0.02* | *0.11* | *4* | *0.01* | *0.39* | *1* | *1* | *0.02* | *0.01* | *3* | *0.27* | *1* | *0.02* | *1* |
|  |  |  |  |  |  |  |  |  |  |  |  |  |  |  |  |  |
| T4 | 0-30 | 7.76 | 0.27 | 0.14 | 26 | 0.02 | 0.64 | 5 | 5 | 0.07 | 0.02 | 26 | 1.05 | 11 | 0.03 | 23 |
|  |  | *0.13* | *0.03* | *0.1* | *4* | *0.01* | *0.41* | *2* | *1* | *0.05* | *0.01* | *5* | *0.65* | *2* | *0.08* | *4* |
|  |  |  |  |  |  |  |  |  |  |  |  |  |  |  |  |  |
|  | 30-60 | 7.79 | 0.21 | 0.14 | 18 | 0.01 | 0.61 | 5 | 4 | 0.02 | 0.02 | 20 | 0.78 | 7 | 0.02 | 7 |
|  |  | *0.13* | *0.04* | *0.11* | *4* | *0.01* | *0.23* | *3* | *1* | *0.02* | *0.01* | *3* | *0.41* | *2* | *0.03* | *2* |
|  |  |  |  |  |  |  |  |  |  |  |  |  |  |  |  |  |
|  | 60-90 | 7.75 | 0.17 | 0.13 | 12 | 0.01 | 0.88 | 4 | 3 | 0.02 | 0.01 | 19 | 0.92 | 5 | 0.02 | 3 |
|  |  | *0.11* | *0.02* | *0.11* | *4* | *0.01* | *0.23* | *1* | *1* | *0.01* | *0.01* | *2* | *0.44* | *2* | *0.03* | *1* |
|  |  |  |  |  |  |  |  |  |  |  |  |  |  |  |  |  |
|  | 90-120 | 7.73 | 0.17 | 0.11 | 11 | 0.01 | 0.86 | 4 | 2 | 0.02 | 0.01 | 20 | 1.07 | 4 | 0.01 | 3 |
|  |  | *0.13* | *0.02* | *0.1* | *3* | *0.01* | *0.28* | *2* | *1* | *0.01* | *0.01* | *2* | *0.46* | *2* | *0.01* | *1* |
| **^†^**Values are the means from twelve replicates with the standard deviation italicized. | | | | | | | | | |  |  |  |  |  |  |  |
| ^‡^Treatments correspond to amounts of Se added via *S. pinnata* (mg Se m^-2^): T0(0), T1(105.4), T2(210.7), T3(421.4), and T4(842.9). | | | | | | | | | | | | | | | | |
| ^¶^Deeper soil sampling took place with highest rates of application: T3 and T4. | | | | | | | | | | | | | | | | |

**Supplementary Table 4. Mean total elemental concentrations in soils amended with *S. pinnata* at postharvest of planting two for carrots and broccoli ^†^.**

|  |  | Total | | | | | | | | | | | | |
| --- | --- | --- | --- | --- | --- | --- | --- | --- | --- | --- | --- | --- | --- | --- |
|  | Depth | B | Ca | Cu | Fe | K | Mg | Mn | Mo | Na | P | S | Zn | Se |
| Treatment^‡^ | (cm) | - - - - - - - - - - - - - - - - - - - - - - - - - - - - - - - - - - - - -- - - -mg kg^-1^ - - - - - - - - - - - - - - - - - - - - - - - - - - - - - - - - - - - - - - | | | | | | | | | | | | |
| T0 | 0-30 | 2 | 2803 | 13 | 16193 | 2495 | 3221 | 288 | 0.4 | 120 | 470 | 69 | 40 | 0.04 |
|  |  | *0.5* | *181* | *1* | *555* | *94* | *140* | *19* | *0.1* | *18* | *68* | *15* | *5* | *0.03* |
|  |  |  |  |  |  |  |  |  |  |  |  |  |  |  |
| T1 | 0-30 | 2 | 2789 | 13 | 16508 | 2531 | 3246 | 295 | 0.4 | 123 | 466 | 67 | 42 | 0.11 |
|  |  | *0.6* | *211* | *2* | *397* | *96* | *108* | *32* | *0.1* | *20* | *62* | *13* | *11* | *0.04* |
|  |  |  |  |  |  |  |  |  |  |  |  |  |  |  |
| T2 | 0-30 | 2 | 2801 | 13 | 16387 | 2508 | 3229 | 287 | 0.4 | 125 | 463 | 73 | 40 | 0.2 |
|  |  | *0.7* | *243* | *1* | *714* | *103* | *130* | *36* | *0.1* | *20* | *49* | *15* | *5* | *0.09* |
|  |  |  |  |  |  |  |  |  |  |  |  |  |  |  |
| T3 | 0-30 | 2 | 2776 | 13 | 16168 | 2504 | 3207 | 292 | 0.4 | 118 | 440 | 66 | 40 | 0.33 |
|  |  | *0.6* | *99* | *1* | *513* | *110* | *152* | *28* | *0.1* | *22* | *31* | *15* | *6* | *0.08* |
|  |  |  |  |  |  |  |  |  |  |  |  |  |  |  |
|  | 30-60 | 1 | 2628 | 13 | 16717 | 2519 | 3267 | 317 | 0.5 | 123 | 463 | 46 | 42 | 0.07 |
|  |  | *0.9* | *164* | *5* | *669* | *111* | *195* | *28* | *0.9* | *12* | *61* | *12* | *9* | *0.03* |
|  |  |  |  |  |  |  |  |  |  |  |  |  |  |  |
|  | 60-90 | 2 | 2563 | 12 | 16630 | 2509 | 3345 | 325 | 0.4 | 125 | 473 | 40 | 37 | 0.05 |
|  |  | *0.6* | *410* | *1* | *692* | *190* | *459* | *36* | *0.2* | *27* | *77* | *8* | *6* | *0.02* |
|  |  |  |  |  |  |  |  |  |  |  |  |  |  |  |
| T4 | 0-30 | 2 | 2679 | 13 | 16167 | 2483 | 3169 | 284 | 0.4 | 117 | 479 | 69 | 40 | 0.55 |
|  |  | *0.4* | *143* | *1* | *578* | *88* | *120* | *29* | *0.2* | *22* | *62* | *15* | *7* | *0.13* |
|  |  |  |  |  |  |  |  |  |  |  |  |  |  |  |
|  | 30-60 | 2 | 2527 | 12 | 16670 | 2515 | 3242 | 306 | 0.3 | 114 | 475 | 48 | 42 | 0.12 |
|  |  | *0.5* | *187* | *2* | *777* | *90* | *162* | *32* | *0.1* | *17* | *87* | *11* | *9* | *0.06* |
|  |  |  |  |  |  |  |  |  |  |  |  |  |  |  |
|  | 60-90 | 2 | 2523 | 11 | 16325 | 2558 | 3338 | 315 | 0.3 | 119 | 489 | 40 | 35 | 0.06 |
|  |  | *1* | *601* | *1* | *696* | *244* | *463* | *29* | *0.1* | *29* | *107* | *11* | *5* | *0.03* |
|  |  |  |  |  |  |  |  |  |  |  |  |  |  |  |
|  | 90-120 | 2 | 2503 | 11 | 16648 | 2569 | 3337 | 318 | 0.3 | 125 | 489 | 40 | 34 | 0.06 |
|  |  | *0.4* | *577* | *1* | *754* | *267* | *504* | *42* | *0.1* | *26* | *95* | *14* | *5* | *0.02* |
| **^†^**Values are the means from twelve replicates with the standard deviation italicized. | | | | | | | | |  |  |  |  |  |  |
| ^‡^Treatments correspond to amounts of Se added via *S. pinnata*  (mg Se m^-2^): T0(0), T1(105.4), T2(210.7), T3(421.4), and T4(842.9). | | | | | | | | | | | | | | |
| ^¶^Deeper soil sampling took place at highest rates of application: T3 and T4. | | | | | | | | | | | | | | |

**Supplementary Table 5. Selenium speciation in soils collected from the short term microplot study conducted on unplanted tiles amended with *S. pinnata*.**

| Day^†^ | SeCyst | MeSeCys | Selenite | SeMet | Selenate | Unknown |
| --- | --- | --- | --- | --- | --- | --- |
| # | - - - - - - - - - - - - - - - - - - % - - - - - - - - - - - - - - - - - - - - - - | | | | | |
|  |  |  |  |  |  |  |
| 1 | 65.7^‡^ | 10 | 4.9 | 1.5 | 0.4 | 17.6 |
|  | (0.8) | (0.6) | (0.6) | (0.4) | (0.2) | (0.6) |
|  |  |  |  |  |  |  |
| 7 | 8.5 | 11.7 | 33.5 | 37.2 | 1.6 | 7.4 |
|  | (2.0) | (3.1) | (7.6) | (7.2) | (1.2) | (1.8) |
|  |  |  |  |  |  |  |
| 15 | 7.7 | 3.9 | 46.6 | 22.3 | 3.8 | 15.8 |
|  | (1.1) | (0.8) | (6.7) | (7.3) | (1.8) | (5.2) |
|  |  |  |  |  |  |  |
| ^†^ Number of days after application of *S. pinnata* (T4) to the soil.  ^‡^ Values represent the mean from six replicates with standard deviation in parenthesis. | | | | | |  |

**Supplementary Table 6. Macro and micronutrient concentrations in broccoli florets and carrots grown in soils amended with *S. pinnata* at planting one**^†^**.**

|  | Total | | | | | | | | | | | | | |
| --- | --- | --- | --- | --- | --- | --- | --- | --- | --- | --- | --- | --- | --- | --- |
| Treatment^‡^ | Cl | B | Ca | Cu | Fe | K | Mg | Mn | Mo | Na | P | S | Zn | Se |
|  | - - - - - - - - - - - - - - - - - - - - - - - - - - - - - - - - - - - - - - - - - µg g^-1^ - - - - - - - - - - - - - - - - - - - - - - - - - - - - - - - - - - - - - - - - | | | | | | | | | | | | | |
|  | Broccoli Florets | | | | | | | | | | | | | |
| T0 | 1681 | 18 | 4671 | 5 | 46 | 26288 | 2246 | 22 | 0.49 | 682 | 6439 | 10450 | 36 | 0.0 |
|  | *276* | *4* | *741* | *1* | *3* | *757* | *133* | *2* | *0.09* | *304* | *397* | *664* | *2* | *0.0* |
|  |  |  |  |  |  |  |  |  |  |  |  |  |  |  |
| T1 | 1404 | 21 | 4681 | 4 | 47 | 26386 | 2299 | 23 | 0.52 | 589 | 6806 | 10782 | 37 | 0.3 |
|  | *139* | *4* | *693* | *1* | *6* | *918* | *205* | *3* | *0.12* | *372* | *899* | *630* | *6* | *0.1* |
|  |  |  |  |  |  |  |  |  |  |  |  |  |  |  |
| T2 | 1467 | 19 | 4616 | 4 | 43 | 27091 | 2216 | 21 | 0.5 | 514 | 6884 | 10505 | 35 | 0.8 |
|  | *219* | *3* | *639* | *1* | *3* | *681* | *70* | *2* | *0.1* | *187* | *539* | *240* | *1* | *0.4* |
|  |  |  |  |  |  |  |  |  |  |  |  |  |  |  |
| T3 | 1543 | 21 | 4593 | 4 | 44 | 27166 | 2167 | 22 | 0.49 | 425 | 6678 | 10451 | 34 | 2.2 |
|  | *194* | *4* | *343* | *2* | *4* | *1483* | *108* | *3* | *0.08* | *197* | *859* | *376* | *3* | *0.9* |
|  |  |  |  |  |  |  |  |  |  |  |  |  |  |  |
| T4 | 1367 | 24 | 4569 | 4 | 46 | 26186 | 2231 | 22 | 0.68 | 541 | 6793 | 10515 | 36 | 7.0 |
|  | *109* | *2* | *258* | *2* | *2* | *446* | *114* | *2* | *0.15* | *281* | *835* | *257* | *3* | *1.3* |
|  | Carrot Roots | | | | | | | | | | | | | |
| T0 | 3995 | 19 | 2007 | 7 | 80 | 26992 | 1105 | 7 | 0.01 | 1016 | 3568 | 1026 | 15 | 0.0 |
|  | *410* | *1* | *159* | *1* | *12* | *1470* | *77* | *1* | *0.02* | *458* | *1226* | *85* | *2* | *0.0* |
|  |  |  |  |  |  |  |  |  |  |  |  |  |  |  |
| T1 | 4341 | 20 | 2071 | 7 | 88 | 27267 | 1098 | 8 | 0.03 | 1194 | 3184 | 993 | 15 | 0.4 |
|  | *394* | *1* | *198* | *1* | *19* | *1291* | *56* | *1* | *0.04* | *526* | *1138* | *66* | *3* | *0.2* |
|  |  |  |  |  |  |  |  |  |  |  |  |  |  |  |
| T2 | 4063 | 19 | 2064 | 6 | 84 | 26705 | 1137 | 8 | 0.04 | 1092 | 3220 | 985 | 12 | 0.7 |
|  | *344* | *2* | *139* | *1* | *24* | *1818* | *77* | *1* | *0.02* | *567* | *1227* | *43* | *2* | *0.1* |
|  |  |  |  |  |  |  |  |  |  |  |  |  |  |  |
| T3 | 4011 | 20 | 2035 | 6 | 86 | 25995 | 1144 | 8 | 0.02 | 969 | 2892 | 1008 | 17 | 2.0 |
|  | *295* | *1* | *141* | *1* | *20* | *674* | *65* | *1* | *0.03* | *304* | *1124* | *81* | *9* | *0.5* |
|  |  |  |  |  |  |  |  |  |  |  |  |  |  |  |
| T4 | 3891 | 21 | 2066 | 7 | 74 | 26935 | 1116 | 8 | 0.03 | 1178 | 3868 | 1088 | 14 | 3.2 |
|  | *375* | *2* | *99* | *1* | *9* | *1506* | *63* | *1* | *0.02* | *405* | *1718* | *71* | *2* | *0.9* |
| **^†^**Values are the means from six replicates with the standard deviation italicized. | | | | | | | | |  |  |  |  |  |  |
| ^‡^ Treatments correspond to amounts of Se added via *S. pinnata* (mg Se m^-2^): T0(0), T1(105.4), T2(210.7), T3(421.4), and T4(842.9). | | | | | | | | | | | | | | |

**Supplementary Table 7. Macro and micronutrient concentrations in broccoli florets and carrots grown in soils amended with S. pinnata at planting two^†^.**

|  | Total | | | | | | | | | | | | | |
| --- | --- | --- | --- | --- | --- | --- | --- | --- | --- | --- | --- | --- | --- | --- |
| Treatment^‡^ | Cl | B | Ca | Cu | Fe | K | Mg | Mn | Mo | Na | P | S | Zn | Se |
|  | - - - - - - - - - - - - - - - - - - - - - - -- - - - - - - -- - - - - - - - µg g^-1^ - - - - - - --- - - - - - - - - - - - - - - - - - - - - - - - - - - - - - - - - - | | | | | | | | | | | | | |
|  | Broccoli Florets | | | | | | | | | | | | | |
| T0 | 2024 | 22 | 4640 | 11 | 71 | 27881 | 2135 | 27 | 1.4 | 878 | 6141 | 11658 | 38 | 0.0 |
|  | *366* | *6* | *1240* | *4* | *16* | *3238* | *375* | *5* | *0.5* | *229* | *657* | *1274* | *5* | *0.0* |
|  |  |  |  |  |  |  |  |  |  |  |  |  |  |  |
| T1 | 1625 | 20 | 4516 | 7 | 75 | 27316 | 2038 | 29 | 1.0 | 1047 | 6228 | 11215 | 36 | 0.5 |
|  | *268* | *5* | *1295* | *1* | *17* | *1815* | *353* | *9* | *0.6* | *322* | *1002* | *1445* | *5* | *0.2* |
|  |  |  |  |  |  |  |  |  |  |  |  |  |  |  |
| T2 | 1942 | 22 | 4223 | 8 | 79 | 28371 | 2074 | 27 | 1.5 | 950 | 6467 | 11700 | 40 | 1.4 |
|  | *451* | *5* | *892* | *2* | *31* | *3431* | *284* | *4* | *1.5* | *192* | *902* | *809* | *5* | *0.4* |
|  |  |  |  |  |  |  |  |  |  |  |  |  |  |  |
| T3 | 1828 | 20 | 4630 | 7 | 65 | 27305 | 2079 | 27 | 1.2 | 1516 | 6006 | 11207 | 34 | 4.0 |
|  | *731* | *5* | *2056* | *1* | *12* | *1036* | *306* | *7* | *1.1* | *523* | *382* | *1732* | *6* | *1.6* |
|  |  |  |  |  |  |  |  |  |  |  |  |  |  |  |
| T4 | 1867 | 19 | 3728 | 8 | 68 | 28577 | 2033 | 25 | 1.2 | 1644 | 6105 | 10511 | 36 | 7.9 |
|  | *183* | *3* | *661* | *3* | *8* | *1696* | *250* | *5* | *0.8* | *296* | *1029* | *1232* | *6* | *1.6* |
|  | Carrot Roots | | | | | | | | | | | | | |
| T0 | 3176 | 24 | 3203 | 8 | 133 | 31371 | 1635 | 13 | 0.1 | 1110 | 4124 | 1189 | 21 | 0.1 |
|  | *498* | *1* | *325* | *2* | *43* | *1861* | *246* | *1* | *0.1* | *517* | *746* | *145* | *2* | *0.1* |
|  |  |  |  |  |  |  |  |  |  |  |  |  |  |  |
| T1 | 2819 | 22 | 3067 | 8 | 154 | 31101 | 1737 | 14 | 0.2 | 853 | 4337 | 1305 | 24 | 0.7 |
|  | *658* | *2* | *166* | *1* | *21* | *2693* | *166* | *2* | *0.2* | *288* | *753* | *154* | *2* | *0.3* |
|  |  |  |  |  |  |  |  |  |  |  |  |  |  |  |
| T2 | 2651 | 24 | 3092 | 8 | 165 | 30454 | 1677 | 13 | 0.2 | 1144 | 4064 | 1305 | 22 | 1.2 |
|  | *505* | *1* | *74* | *1* | *33* | *1132* | *141* | *2* | *0.1* | *400* | *607* | *126* | *2* | *0.4* |
|  |  |  |  |  |  |  |  |  |  |  |  |  |  |  |
| T3 | 2671 | 24 | 3097 | 12 | 127 | 32961 | 1582 | 12 | 0.1 | 1141 | 4434 | 1403 | 25 | 3.1 |
|  | *742* | *2* | *235* | *11* | *33* | *3558* | *122* | *1* | *0.1* | *362* | *573* | *97* | *5* | *1.0* |
|  |  |  |  |  |  |  |  |  |  |  |  |  |  |  |
| T4 | 2710 | 21 | 3072 | 8 | 139 | 31660 | 1621 | 12 | 0.3 | 1117 | 4125 | 1392 | 20 | 6.3 |
|  | *242* | *1* | *216* | *1* | *32* | *2044* | *89* | *1* | *0.1* | *320* | *753* | *138* | *3* | *1.0* |
| **^†^**Values are the means from six replicates with the standard deviation italicized. | | | | | | | | | |  |  |  |  |  |
| ^‡^ Treatments correspond to amounts of Se added via *S. pinnata* (mg Se m^-2^): T0(0), T1(105.4), T2(210.7), T3(421.4), and T4(842.9). | | | | | | | | | | | | | | |

**Supplementary Table 8. Structure matrix (pooled with canonical structure) and function at group centroid for 0-5 and 5-30 cm depths.**

|  | Structure Loadings | | | |
| --- | --- | --- | --- | --- |
|  | 0-5 cm | | 5-30 cm | |
| Variable | CV 1 | CV 2 | CV 1 | CV 2 |
| Fungi | 0.58 | 0.64 | -0.16 | 0.55 |
| AMF | 0.5 | 0.36 | 0.12 | 0.39 |
| Gram + | 0.49 | 0.51 | 0.01 | 0.35 |
| Gram - | 0.6 | 0.45 | 0.04 | 0.28 |
| Actino | 0.25 | 0.46 | 0.01 | 0.41 |
| Eukaryote | -0.09 | 0.51 | 0.12 | 0.42 |
|  | Group Centroids | | | |
| Broccoli T4^†^ | -0.35 | 0.29 | -0.08 | 0.45 |
| Broccoli T0+ | -0.84 | -0.17 | 0.76 | -0.53 |
| Carrot T4^†^ | 0.61 | 0.6 | -1.34 | -0.19 |
| Carrot T0+ | 0.57 | -0.72 | 0.66 | 0.27 |
| ^†^T4 is treatment 4 and T0 is control, as described in materials and methods. | | | | |

## Supplementary Figure

## Supplementary Figure 1. Total water soluble Se distribution in soil amended with *S. pinnata (T4)* at postharvest of planting two for broccoli and carrots (values represent the mean from six replicates and standard deviation bar).

**Supplementary Figure 2.** Water soluble Se speciation distribution in soil amended with *S. pinnata* (T4) at postharvest of planting two for broccoli and carrot (values represent the mean from six replicates and standard deviation bar).


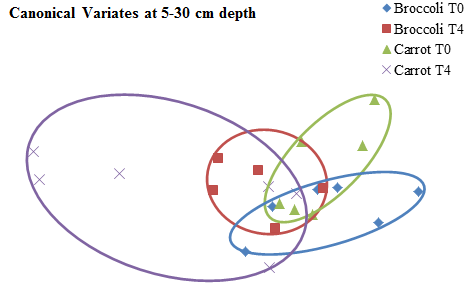

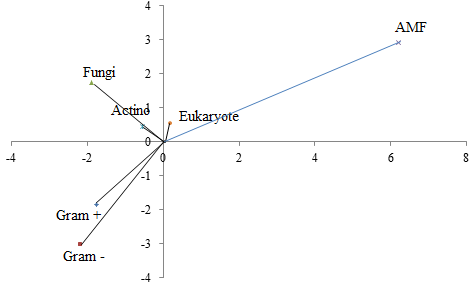


**Supplementary Figure 4**. Canonical multivariate analysis of variance of phospholipid fatty acid biomarkers for 5-30 cm depth. Vectors represent standardized canonical coefficients and indicate the relative contribution of each biomarker group to each canonical variate. Six soil samples were collected, respectively from application rate (T4) and control (T0) soils growing broccoli and carrots (as described in materials and methods).


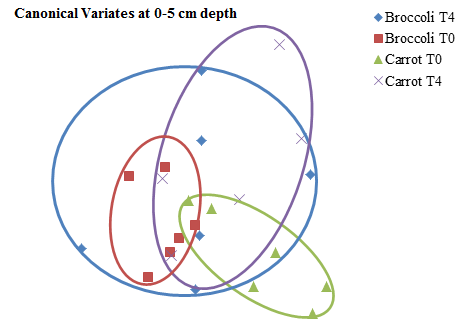

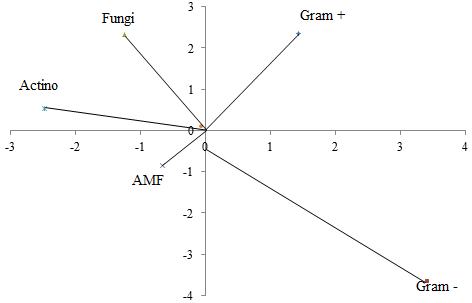


**Supplementary Figure 3**. Canonical multivariate analysis of variance of phospholipid fatty acid biomarkers for 0-5 cm depth. Vectors represent standardized canonical coefficients and indicate the relative contribution of each biomarker group to each canonical variate. Six soil samples were collected, respectively, from application rate (T4) and control (T0) soils growing broccoli and carrots (as described in materials and methods).
